# Particle Filter Based Monitoring and Prediction of Spatiotemporal Corrosion Using Successive Measurements of Structural Responses

**DOI:** 10.3390/s18113909

**Published:** 2018-11-13

**Authors:** Sang-ri Yi, Junho Song

**Affiliations:** Department of Civil and Environmental Engineering, Seoul National University, Seoul 08826, Korea; yisangri@snu.ac.kr

**Keywords:** Bayesian filter, Bayesian inversion, corrosion, Karhunen-Loève expansion, Markov Chain Monte Carlo (MCMC) simulation, particle filter, structural deterioration

## Abstract

Prediction of structural deterioration is a challenging task due to various uncertainties and temporal changes in the environmental conditions, measurement noises as well as errors of mathematical models used for predicting the deterioration progress. Monitoring of deterioration progress is also challenging even with successive measurements, especially when only indirect measurements such as structural responses are available. Recent developments of Bayesian filters and Bayesian inversion methods make it possible to address these challenges through probabilistic assimilation of successive measurement data and deterioration progress models. To this end, this paper proposes a new framework to monitor and predict the spatiotemporal progress of structural deterioration using successive, indirect and noisy measurements. The framework adopts particle filter for the purpose of real-time monitoring and prediction of corrosion states and probabilistic inference of uncertain and/or time-varying parameters in the corrosion progress model. In order to infer deterioration states from sparse indirect inspection data, for example structural responses at sensor locations, a Bayesian inversion method is integrated with the particle filter. The dimension of a continuous domain is reduced by the use of basis functions of truncated Karhunen-Loève expansion. The proposed framework is demonstrated and successfully tested by numerical experiments of reinforcement bar and steel plates subject to corrosion.

## 1. Introduction

Prediction of structural deterioration has intrinsic difficulties originating from erroneous assumptions in the deterioration progress model and uncertainties, and temporal changes in environmental conditions. To alleviate errors in the prediction, measurement data could be used to adjust the predicted states or improve the models used for predictions. Such data can be obtained from real-time monitoring of the structure or periodic field inspections. However, these measurement data may contain various degrees of signal noises and often deterioration states of a structure are not directly measurable due to physical, operational or technical restrictions. In addition, measurement data are usually obtained as a time series. Therefore, there is a pressing need for a probabilistic framework to assimilate successive measurements and numerical deterioration progress models [1,2,3,4].

In efforts to address this need, recursive Bayesian updating methods—often termed “Bayesian filter” methods—such as the Kalman filter, extended Kalman filter and particle filter, gained popularity as a tool for the prognosis and diagnosis of structural deterioration phenomena [5]. In particular, the Kalman filter and extended Kalman filter, which can provide the analytical solutions of the linear Gaussian Bayesian filter, were extensively applied to track fatigue and corrosion propagation of structural elements [6,7,8,9]. On the other hand, the particle filter approach, which is a simulation based empirical solution of the Bayesian filter, gained subsequent but remarkable spotlight in recent years [10,11,12,13]. This is mainly due to its wide applicability to various model structures and also drastic improvement of computational capabilities, which now enables real-time estimations [14,15,16]. Especially, particle filter algorithms are able to update not only the system states but also the probability distributions of parameters in the equations representing the system and measurement process, without tailored re-formulations [17]. Therefore, including online parameter identification [15,18,19] as a part of particle filter applications has been widely practiced. In this paper, we propose utilizing this parameter-updating feature of the particle filter for the purpose of monitoring and prediction of structural deterioration. The applicability of the proposed framework is tested in terms of a wide range of parameters. These include stochastic parameters that represent the degree of variability of the environmental uncertainty and dynamic characteristics to illustrate a variety of situations in which the mathematical formulation of the deterioration progress model does not accord with actual phenomena.

An important but challenging task in the prognosis of deterioration is the probabilistic inference of spatiotemporal patterns of structural deterioration using sparsely measured indirect observations such as structural responses. Much research has been carried out to deal with this damage detection problem using advanced optimization methods [20,21,22,23]. There were also a few attempts to identify the locations of damage as well as its evolutions using the Bayesian filter framework. However, these attempts focused on the dynamics of a single crack growth, rather than global pattern of structural deterioration [19] or were limited to truss-type structures [7]. In this paper, we propose a method to predict spatiotemporal deterioration pattern in a continuous domain by using only a finite number of measurements of structural responses, for example strains and displacements at sensor locations. The method requires an inverse structural analysis to infer deterioration states, for example thickness of steel plate, from evidence of structural response, for example displacement and strain. For this purpose, a Bayesian inversion based random field updating is incorporated. To reduce the dimension of continuous domain of interest, we employ a truncated Karhunen-Loève (KL) expansion. This kind of combination of Bayesian inversion and KL-expansion has been employed in several research works for inverse analysis of continuous domain [24,25,26] but not along with Bayesian filter framework or to predict structural deterioration.

In the following section, we briefly present general backgrounds of Bayesian filter and particle filter approach, and proposes a particle filter based algorithm as a probabilistic framework for deterioration monitoring and prediction. In Section 3, Bayesian inversion-based measurement model is presented in detail to relate sparsely measured structural response and corresponding deterioration states. Numerical demonstrations are provided in Section 4 in which the proposed method is applied to monitoring and prediction of the spatiotemporal evolution of corrosion on reinforcement bars and steel plate elements, respectively. Especially, state-parameter updating capabilities of the proposed method are investigated in detail by introducing parameters representing the stochastic nature of the environmental conditions and time-varying characteristics. Finally, Section 5 provides a summary and future research topic. 

## 2. Particle Filter Based Monitoring and Prediction of Structural Deterioration

### 2.1. Brief Review of Bayesian Filter Approach

This section briefly introduces the concept of Bayesian filter to provide background information of the proposed framework. In principle, a Bayesian filter estimates and updates the joint probability distribution of the states and model parameters of interest by aggregating past measurement history obtained up to the current time point. Under successive measurement conditions, the real-time updating of the joint probability density can be conducted by recursively performing two step-procedures: *propagation* and *updating* steps. The propagation step describes temporal evolution of the system and propagation of system uncertainty and the estimation result endures until new measurements are obtained. As observations are acquired at a certain time instance, the updating step is activated to incorporate the additional information embedded in the measurement into the state via Bayes’ theorem. Therefore, to formulate Bayesian filter problem, we need to predefine two models, that is system (propagation) model and measurement model, which are described respectively as

(1a) $$x_{k+1}=f\left(x_{k},\theta_{k},w_{k}\right)$$

(1b) $$y_{k}=h\left(x_{k},v_{k}\right)$$

where k is the time index, $x_{k}$ is the vector of the states of interest, for example deterioration state of structure, at time $t_{k},$ $\theta_{k}$ is the vector of system model parameters and $y_{k}$ is the vector of the measured quantities. The functions f(·) and h(·) respectively denote the system model and measurement model. The variables $w_{k}$ and $v_{k}$ represent the uncertainties in the corresponding models.

In the propagation step, the temporal evolution of the states is probabilistically described using a dynamic system model. The propagation of the system state is described in terms of transition probability density function $p\left(x_{k}|x_{0:k-1},\theta_{0:k-1}\right),$ where $x_{0:k-1}=\left\{x_{0},x_{1},\ldots ,x_{k-1}\right\}$ and $\theta_{0:k-1}=\left\{\theta_{0},\theta_{1},\ldots ,\theta_{k-1}\right\}$, or, by making the Markov process assumption, $p(x_{k}|x_{k-1},\theta_{k-1})$. In the updating step, as measurements $y_{k}$ at time $t_{k}$ are acquired, the joint probability density function is updated via Bayes’ theorem, that is,

(2) $$p\left(x_{k},\theta_{k}|y_{k}\right)=c\cdot L\left(y_{k}|x_{k},\theta_{k}\right)\cdot p\left(x_{k},\theta_{k}\right)$$

where $L(y_{k}|x_{k},\theta_{k})$ is the likelihood of the measurement based on Equation (1b), $p\left(x_{k},\theta_{k}\right)$ is the prior distribution obtained from the propagation step and c is the normalizing constant. Note that Equation (2) employs the Markov process assumption by using $y_{k}$ instead of $y_{0:k}$ [17].

The closed-form solutions of both propagation and updating step can be derived if the two models f(·) and h(·) in Equation (1) are linear and the related uncertainties in $x_{0}$, $w_{k}$ and $v_{k}$ are Gaussian. The recursive process of these closed-form calculations is referred to as *Kalman filter*. However, when either one of the conditions is not satisfied, as often seen in general applications in structural deterioration monitoring and prediction problem, solving a Bayesian filter requires introducing approximation- or simulation-based recursive algorithms. Such algorithms include extended Kalman filter, unscented Kalman filter and particle filter.

### 2.2. Particle Filter Based Prediction of Structural Deterioration

Particle filter solves Bayesian filter problems by means of simulations and therefore, is also known as a sequential Monte Carlo method. Particle filter is useful especially when the problem includes highly nonlinear or complicated system models and/or non-Gaussian distributions. The joint probability density function (PDF) of system states and model parameters is approximated by the empirical distribution represented by a set of sample realizations, that is

(3) $$\hat{p}\left(X\right)\approx \frac{1}{N}\overset{N}{\underset{i=1}{\sum }}\delta \left(X-X^{i}\right)$$

in which $\hat{p}\left(X\right)$ denotes the empirical distribution model of the states and parameters X={x, θ}, represented by the corresponding set of realized values termed “particles” $X^{i}=\left\{x^{i},\;\theta^{i}\right\}$ where i denotes the index of the particles (i=1,2,…,N). The state transition in the propagation step is simulated directly by substituting the particles into Equation (1a) and therefore, particle filter naturally allows us to engage nonlinear deterioration models.

**Figure 1:**
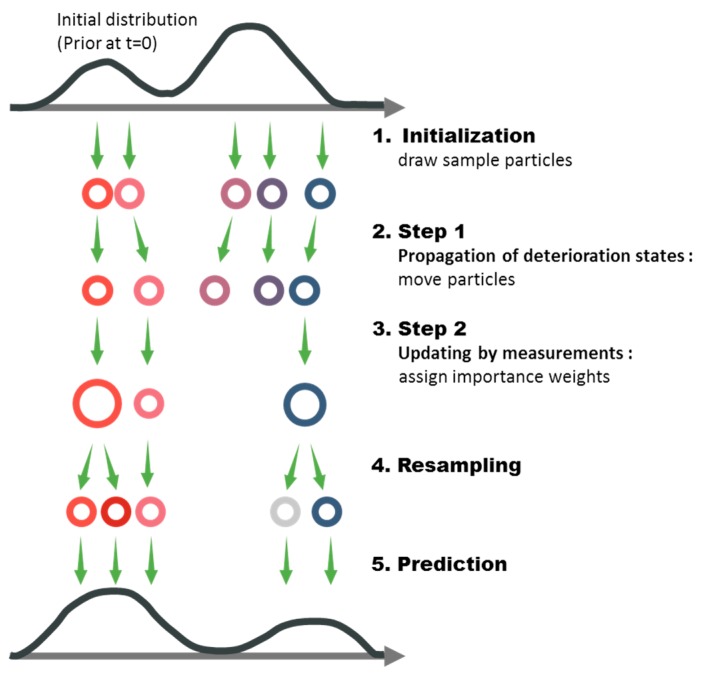
Procedure of particle filter using SIR algorithm.

For the updating step in Equation (2), there are several approaches of Bayesian updating. One of the classical approaches is to use sequential importance resampling (SIR) method, whose procedure is illustrated in Figure 1. Below is a summary of the procedure of particle filter using SIR algorithm, with the system (deterioration) state $x_{k}$ and static and dynamic model parameters $\theta_{k}=\left\{\theta_{k,s},\theta_{k,d}\right\}$ [27].

1. Initialization: N-number of samples, or particles, of state and parameters $\left\{x_{0}^{i},\theta_{0}^{i}\right\}$, are generated from the PDFs representing the initial knowledge. The initial weights of the samples are set equal to each other. Afterward, the following two steps are repeated.
2. Step 1—Propagation of deterioration states: At time step (k−1), the temporal progress of deterioration at the next time step k can be simulated using the system equation for individual particles, that is
  (4) $$x_{k}^{i}=f\left(x_{k-1}^{i},\theta_{k-1}^{i},w_{k-1}^{i}\right)$$
  where $w_{k-1}^{i}$ is randomly generated noise representing the uncertainty in the propagation model. While the static model parameters stay the same, that is $\theta_{k,s}^{i}=\theta_{k-1,s}^{i}$, the dynamic model parameters will propagate by $\theta_{k,d}^{i}=g\left(\theta_{k-1,d}^{i}\right)$, where g(·) is a physical or mathematical evolution function of parameter introduced in the given problem for such dynamic model parameters.
3. Step 2—Updating by measurements: As noisy measurements $y_{k}$ are obtained at time step k, the likelihood of each particle can be attained from Equation (1b). The importance weight of each particle is then calculated as proportional to its likelihood as follows:
  (5) $$q_{k}^{i}=\frac{L\left(y_{k}|x_{k}^{i},\theta_{k}^{i}\right)}{\sum_{j=1}^{N}L\left(y_{k}|x_{k}^{j},\theta_{k}^{j}\right)}$$
  Then the posterior distribution at time step k is described in terms of specific values of particles and their weights, which respectively represent the prior distribution and the likelihoods. The next task is to resample the particles based on the weights from Equation (5), by duplicating the particles with higher importance weight and eliminating the particles having low importance weight. In this resampling task, new particle sets $\left\{x_{k}^{i+},\theta_{k}^{i+}\right\}$, i=1,2,…,N, are sampled from the multinomial distribution of current particles with their selection probability:
  (6) $$\mathrm{Pr}\left(\left\{x_{k}^{i+},\theta_{k}^{i+}\right\}=\left\{x_{k}^{n},\theta_{k}^{n}\right\}\right)=q_{k}^{n},n=1,2,\ldots ,N$$
  The resampled particles again possess the equal weight and thus represent the posterior distribution of the parameters. This *resampling procedure* includes trimming outlier scenarios to resolve the divergence problem but can result in *particle depletion*, that is, diversity of particles can be lost and few survived particles represent the whole distribution. This can be severe especially when the propagation noise is small or particle carries static parameters. To address the issue, in this research, states and parameters are subjected to arbitrary artificial evolution after each resampling so that the resulting lost information is properly compensated by scheme of shrinkage, that is, samples are pushed toward their means before adding artificial noise [28].
4. Prediction: For the purpose of predicting the states for future time durations in which no observations are available, only Step 1 is repeated until the time index reaches the end point of the future time duration of interest. The means or medians of the empirical distributions can be used as estimates of future states and parameters.

Since particle filter approach relies on sampling and empirical updating, larger simulation cost is required compared to other Bayesian filters such as Kalman filter and variations of unscented Kalman filter. Therefore, the particle filter approach may not be applicable to real-time simulations of a short-term dynamic events, especially when simulations of involving models are time-consuming. However, since structural deterioration is a phenomenon that develops slowly over a long period of time, the computing time is not an issue. Besides, the particle filter instead has a useful advantage in its flexibility regarding types of system models and probability distributions. Owing to its sampling-based approach, the particle filter is able to incorporate complex and highly nonlinear models to describe the system, as well as non-Gaussian distributions of errors and uncertainties without using tailored modifications. Noting that direct measurement of deterioration states is often infeasible in practice, particle filter seems to possess a clear advantage in that a wide range of inspection schemes can be incorporated. For example, measurement of structural responses, for which the measurement equation would be an inverse structural analysis, can also be embraced as demonstrated in the numerical examples. Another important advantage of applying particle filter to the deterioration problem is that uncertainties in the system model parameters, which is difficult to estimate or quantify in advance, can be reduced by its parameter-updating capability.

### 2.3. Challenges in Application of Particle Filter to Spatial Pattern of Deterioration

To predict the deterioration of a structural element that has continuous domain, a uniformly discretized domain space could be considered so that the deterioration progress of each spatial nodal point is tracked and predicted individually by particle filter approach. In this approach, the measurements of deterioration states are required at every individual node. As the domain of interest grows larger or one wishes to increase the spatial resolution of prediction, the number of nodes will also increase and it becomes challenging to obtain measurements covering the whole domain. Also there are situations in which direct measurements of deterioration state are not available due to physical restrictions but instead, other indirect information is obtainable. In this research, we consider situations in which measurements of structural response under loading tests are available as indirect information. Therefore, the approach should include a process to transform the sparsely measured quantity, such as strain measurements in locations where sensors are installed, to the full-domain description of deterioration state such as degradation of effective thickness or elastic modulus. It can be considered an inverse problem of identifying system property from structural responses. For this purpose, a Bayesian inversion method is introduced along with a random field representation of spatial pattern of deterioration [24]. The next section presents the Bayesian inversion method developed to identify the spatial pattern of deterioration using the particle filter approach.

## 3. Particle Filter Based Monitoring and Prediction Using Sparse Mechanical Measurements

### 3.1. KL-Expansion-based Representation of Spatial Pattern of Deterioration

Consider a domain of interest, for example the surface of a structural member, $\Omega \subset {\mathbb{R}}^{2}$. Let us introduce a Gaussian random field x(z), where z∈Ω, that represents the uncertain deterioration state continuously distributed in the domain. In this setting, the actual deterioration state is interpreted as one of the possible realizations of this random field. By using a random-field discretization approach based on basis functions, we can describe the state in the continuous domain using a finite number of parameters which are independent of domain size and resolution. Then we aim to update the probability distributions of the random field parameters based on evidences using Bayes’ theorem and find optimal estimate of deterioration field from the posterior distributions.

Among available discretization methods [29], this study uses a spectral representation method termed, Karhunen-Loève expansion (KL-expansion), which describes the Gaussian random field x(z) as

(7) $$x\left(z\right)=\mu_{x}\left(z\right)+\overset{\infty }{\underset{m=1}{\sum }}\sqrt{\lambda_{m}}\varphi_{m}\left(z\right)\xi_{m}$$

where $\mu_{x}\left(z\right)$ is the deterministic mean function of the random field, $\left\{\xi_{m}\right\}$ are independent standard normal random variables that describe the stochastic contributions of the pre-specified basis functions. We will refer to these variables as *KL-random variables* and denote them by a vector form $\xi =\left\{\xi_{m}\right\}$. $\lambda_{m}$ and $\varphi_{m}\left(z\right)$ in Equation (7) are deterministic scaling factor and basis function of the random field respectively, which are obtained by solving the Fredholm equation of second kind [30]:

(8) $$\int_{D}C\left(z_{1},z_{2}\right)\varphi_{m}\left(z_{2}\right)dz_{2}=\lambda_{m}\varphi_{m}\left(z_{1}\right)$$

where $C\left(z_{1},z_{2}\right)$, $z_{1},z_{2}\in \Omega$, is the autocovariance function introduced to characterize the random field. Note that Equation (8) is an eigenvalue problem in which $\lambda_{m}$ is positive and finite eigenvalue and $\varphi_{m}\left(z\right)$ is eigenfunction having orthogonality property. This equation is derived from spectral decomposition of the covariance function, that is,

(9) $$C\left(z_{1},z_{2}\right)=\overset{\infty }{\underset{m=1}{\sum }}\lambda_{m}\varphi_{m}\left(z_{1}\right)\varphi_{m}\left(z_{2}\right)$$

Refer to [30] for more detailed explanations and numerical methods to solve Equation (8). From Equation (7), one can approximate the random field by using only a set of basis functions that have significant scaling values. Under homogeneous assumptions $\mu_{x}\left(z\right)=\mu_{x}$, the following approximate form of a truncated KL-expansion is obtained:

(10) $$x\left(z\right)≅\hat{x}\left(z\right)=\mu_{x}+\overset{M}{\underset{m=1}{\sum }}\sqrt{\lambda_{m}}\varphi_{m}\left(z\right)\xi_{m}$$

### 3.2. Bayesian Inference of KL-Random Variables as Measurement Model in PF

**Figure 2:**
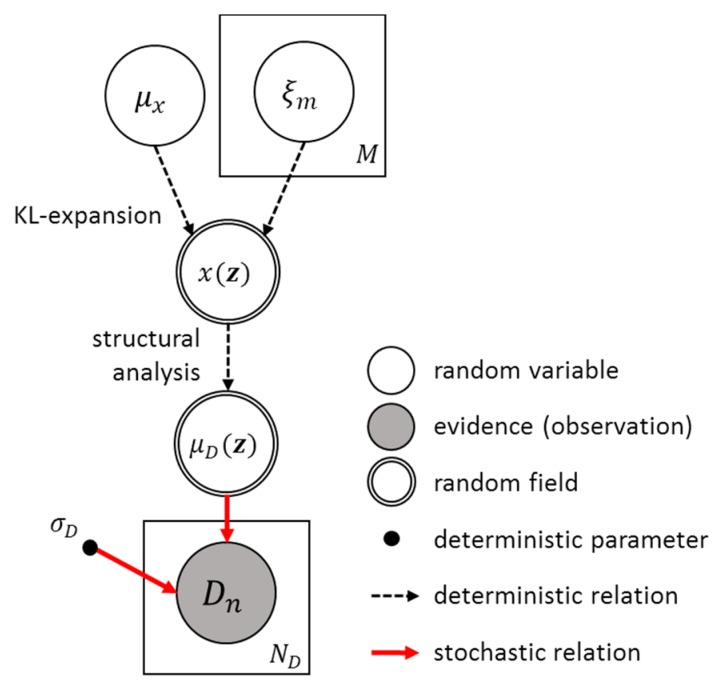
Graphical representation of the measurement model used for Bayesian inversion.

From Equation (10), it is noted that, for a presumed autocovariance function of deterioration, the specification of $\mu_{x}$ and ξ will automatically lead to the full-domain description of the deterioration state $\hat{x}\left(z\right).$ These variables can be estimated and updated by Bayes’ rule using available measurements as evidences. The prior distribution of KL-random variables can be selected as independent standard normal variables and the prior mean of random field can be retrieved based on deterioration model without loss of generality. As observations of structural response, $D^{obs}=\left\{D_{n}^{obs}\right\},\;n=1,\ldots ,N_{D}$, are acquired at sensors located in domain, the posterior probability is obtained as

(11) $$p\left(\mu_{x},\xi |D^{obs}\right)=c\cdot L\left(D^{obs}|\mu_{x},\xi \right)\cdot \;p\left(\mu_{x},\xi \right)$$

where c denotes the normalizing constant and $L\left(D^{obs}|\mu_{x},\xi \right)$ is the likelihood function. By modelling the error of sensor as unbiased independent Gaussian random variables having standard deviation of $\sigma_{D}$, the observation at the n-th location, $D\left(z_{n}\right)$ and the likelihood of all observation points, $L\left(D^{obs}|\mu_{x},\xi \right)$ are described as follows respectively:

(12a) $$D\left(z_{n}\right)=h_{KL-FEM}\left(z_{n};\mu_{x},\xi \right)+\sigma_{D}\varepsilon_{n}$$

(12b) $$L\left(D^{obs}|\mu_{x},\xi \right)=\overset{N}{\underset{n=1}{\prod }}L(D_{n}^{obs}|\mu_{x},\xi )=\overset{N}{\underset{n=1}{\prod }}f_{N}\left(D\left(z_{n}\right)=D_{n}^{obs}|\mu_{D,n},\sigma_{D}^{2}\right)$$

where $h_{KL-FEM}\left(z_{n};\mu_{x},\xi \right)$ is the deterministic measurement function that relates the measured structural responses and the states at the location $z_{n}$ for given KL-random variables ξ and $\mu_{x}$. This function includes the composition of KL-random variables shown in Equation (10) and structural analysis, for example finite element analysis using the constructed field to obtain the corresponding response. The random variable $\varepsilon_{n}\sim N\left(0,1^{2}\right)$ represents the uncertainty in the measurement noise. The function $f_{N}\left(D\left(z_{n}\right)|\mu_{D,n},\sigma_{D}^{2}\right)$ in Equation (12b) represents the Gaussian probability density function of $D\left(z_{n}\right)$ with the mean $\mu_{D,n}=\mu_{D}\left(z_{n}\right)=h_{KL-FEM}\left(z_{n};\mu_{x},\xi \right)$ and the standard deviation $\sigma_{D}$. Figure 2 illustrates the Bayesian parameter updating process described in Equation (11).

However, the updating in Equation (11) is a challenging task because of the difficulty of the integration involved in the evaluation of the normalizing constant and parameters of the posterior distribution. To overcome the difficulty, Markov Chain Monte Carlo (MCMC) sampling method is adopted in this study to obtain the empirical posterior probability density function through samples, that is, without performing the numerical integration [31]. The application of MCMC sampling to Equation (11) only requires the knowledge of prior distribution and the calculation of likelihood given $\mu_{x}$ and ξ, (involving forward structural analysis) and does not need to compute the normalizing constant. From the sample means of the empirical posterior distributions, we obtain estimates of the KL-random variables, that is

(13a) $${\hat{\xi }}_{m}=\int_{-\infty }^{\infty }p\left(\xi_{m}|D^{obs}\right)\xi_{m}d\xi_{m}\approx \frac{1}{N_{\xi }}\overset{N_{\xi }}{\underset{j=1}{\sum }}\xi_{m}^{j}$$

(13b) $${\hat{\mu }}_{x}=\int_{-\infty }^{\infty }p\left(\mu_{x}|D^{obs}\right)\mu_{x}d\mu_{x}\approx \frac{1}{N_{\xi }}\overset{N_{\xi }}{\underset{j=1}{\sum }}\mu_{x}^{j}\;$$

where $N_{\xi }$ is the number of MCMC samples. Substituting these KL-random variable estimates into Equation (10), the spatial distribution of the deterioration state is inferred as

(14) $$\hat{x}\left(z\right)={\hat{\mu }}_{x}+\overset{M}{\underset{m=1}{\sum }}\sqrt{\lambda_{m}}\varphi_{m}\left(z\right){\hat{\xi }}_{m}$$

**Figure 3:**
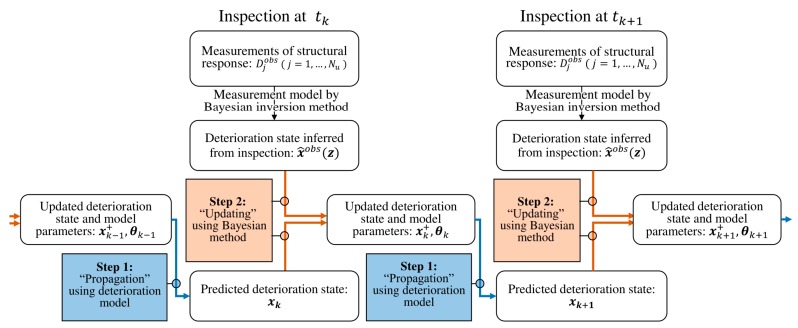
Recursive particle filter-based framework to monitor and predict spatiotemporal progress of structural deterioration.

Now returning to the particle filter framework, it is noted that the function $h_{KL-FEM}\left(\cdot \right)$ is the measurement model h(·) in Equation (1b). The measurement quantity $y_{k}$ in Equation (1b) corresponds to the mechanical observations $D^{obs}$ at the time step k, and $x_{k}$ is the deterioration state of the locations of interest x(z) at time step k. Figure 3 illustrates the recursive procedure of the particle filter framework proposed to predict spatial pattern of deterioration based on sparsely measured structural responses.

## 4. Numerical Investigations

### 4.1. Corrosion of Reinforcing Bar

To illustrate the applicability of particle filter approach to monitoring and prediction of deterioration and its parameter-updating capability, we first consider a numerical example in which direct measurements can be gained from a non-destructive test (NDT) device. The corrosion of reinforcing bar is chosen as the deterioration phenomenon of interest. Although other general models could be used with the proposed PF-based approach, two simple corrosion and measurement models are employed in this example.

#### 4.1.1. System Model: Corrosion Progress

A simple corrosion progress model of reinforcing bar is adopted from a report by Federal Emergency Management Agency [32]. The model describes the loss of the effective cross-section area due to corrosion using the following state-space form:

(15) $$A_{n+1}=A_{n}-P^{2}\Delta t$$

where $A_{n}$ is the effective cross-sectional area of reinforcing bar at *n*-th year, P is the annual penetration rate in unit of length per year and Δt is time duration in unit of year. The original assumptions in Equation (15) include: (a) the annual loss of the cross-sectional area of a rebar is the square of annual penetration rate, (b) temperature effect is not considered and (c) the annual penetration rate is known and static. In this numerical example, the last assumption is relaxed, that is the penetration rate is considered to be uncertain and dynamic.

#### 4.1.2. Measurement Model: NDT Test to Measure the Percentage Loss of the Cross-Section

Suppose an inspection device provides successive measurements of a quantity that has an explicit relationship with the target deterioration state. For example, an induced magnetic field (IMF) device [33,34] can measure the induced magnetic field along the length of reinforcing bar and the following straightforward equation gives the corresponding percentage loss of the cross-section area by corrosion, that is the target deterioration state:

(16) $$\mathrm{percentage}\;\mathrm{loss}\;\mathrm{of}\;\mathrm{the}\;\mathrm{cross}-\mathrm{section}=\frac{A_{corr}}{A_{0}}=\left(1-\frac{B_{corr}-B_{b}}{B_{healthy}-B_{b}}\right)$$

where $A_{0}$ and $A_{corr}$ respectively are the initial cross-sectional area and intact area of cross-section after corrosion evolution, $B_{corr}$ is the field test value of induced magnetic field in unit gauss (G), $B_{healthy}$ is the healthy value and $B_{b}$ is the baseline value that can be obtained with no steel reinforcing bar present. 

#### 4.1.3. Generation of Reference State and Problem Setting

**Figure 4:**
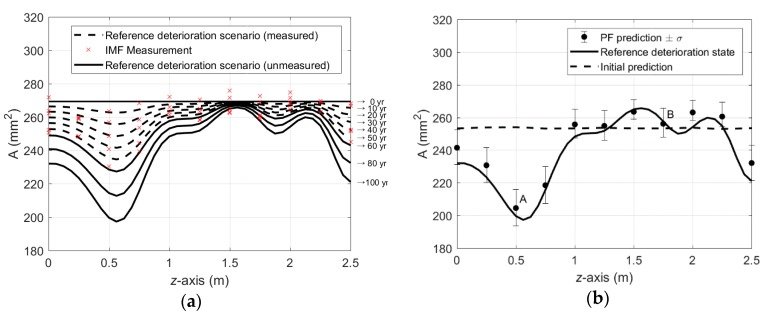
Generated reference scenario and prediction by particle filter approach: (**a**) Reference deterioration scenario and noisy periodic measurements; (**b**) Prediction by particle filter approach for 100th year of the operation.

To generate a reference scenario of corrosion, the penetration rate at each location along the bar is generated via numerical realization of assumed stochastic distributions. The penetration rate, with unit of mm/year, is assumed as a dynamic parameter whose value gradually increases linearly with additive random disturbances, that is $P\left(z,t\right)=P_{0}\left(z\right)+P_{1}\left(z\right)t+\varepsilon_{P}\left(z,t\right)$. Here, the two model coefficients are randomly generated by $P_{0}\left(z\right)\sim N\left(0.6,0.4^{2}\right)$ and $P_{1}\left(z\right)\sim N\left(0.00075,0.0002^{2}\right)$ to account for the spatial variations of penetration rates, while the last term $\varepsilon_{P}\left(z,t\right)\sim N\left(0,0.003^{2}\right)$ additionally describes randomness in temporal variations. Each of the three parameters is assumed to have spatial correlation expressed by following exponential autocovariance function:

(17) $$\;C\left(z_{1},z_{2}\right)=\sigma^{2}\exp\left(-\frac{∥z_{1}-z_{2}∥}{L_{d}}\right)$$

where $z_{1}$ and $z_{2}$ refer to coordinates of two different locations in the domain and each of their correlation length is $L_{d}=1\;m$. Note that the autocovariance function in Equation (17) is given in terms of vector coordinates to represent general dimensions but for this particular example, we use scalar coordinates $z_{1}$ and $z_{2}$ to denote locations in the reinforcement bar. While the spatial correlation of $\varepsilon_{P}\left(x,t\right)$ holds, it is assumed to be temporally uncorrelated in this example. As a result, the cross-section area is decreased over time following Equation (15) with the randomly generated penetration rates. The curves in Figure 4a show a reference scenario of corrosion progress generated for the period of 100 years. A set of IMF scan measurements is simulated by adding artificial non-biased noise to the corrosion scenario. The relative error in the IMF measurements are assumed to be 3% upon healthy state and the simulated IMF measurements are visualized by cross-markers in Figure 4a. It is assumed that periodic full-domain measurements with 10-year intervals are provided until the 50th year of the operation. 

#### 4.1.4. Results and Discussions

**Figure 5:**
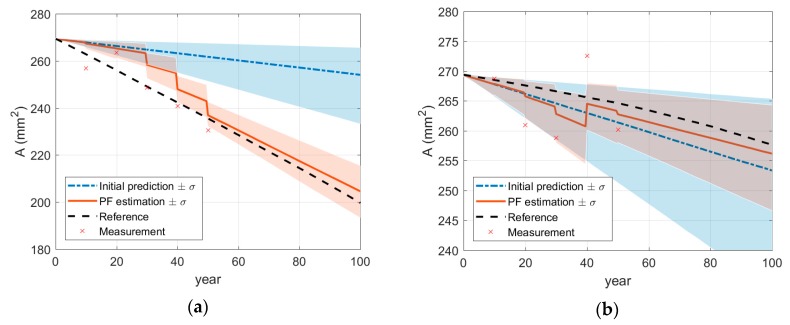
Monitoring and prediction of cross-section by particle filter at specific locations: (**a**) at point A; (**b**) at point B.

**Figure 6:**
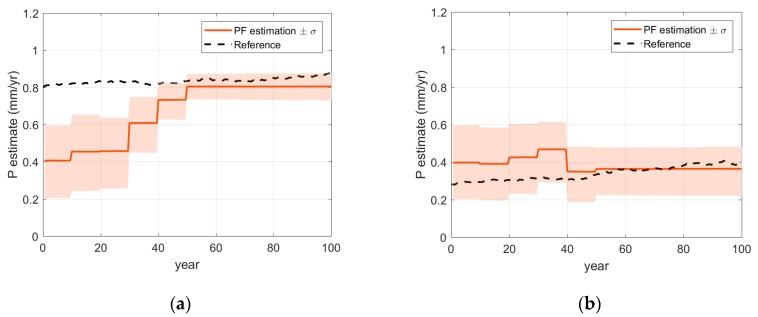
Updating of corrosion model parameters by particle filter at specific locations: (**a**) *P* at point A; (**b**) *P* at point B.

Imposing initially inaccurate knowledge of homogeneous penetration rate, P=0.4 mm/year, the effective areas along the reinforcing bar are predicted for the 100th year of the operation using two methods: (a) the model in Equation (15) with the initial penetration rate and (b) the PF with the noisy periodic measurements up to 50th year. The comparison in Figure 4b shows that, even though the measurement error was not trivial, the prediction results by the PF are fairly reasonable compared to a poor prediction by the model with the initial penetration rate. For detailed investigation, at two locations A and B in Figure 4b, the time histories of monitoring (up to 50 years) and prediction (afterwards) are presented in Figure 5. The results show that the monitoring results are gradually improved as more measurements are acquired. Figure 6a,b confirm that the parameter-updating capability of PF utilizes the measurement data to track down the penetration rate near to the reference value, which enables accurate prediction for future time duration.

### 4.2. Prediction of Spatial Corrosion Pattern of Steel plate

Next, the whole framework proposed in this paper is applied to monitor and predict the spatial evolution of pitting corrosion in a steel plate member of dimensions 2.5 × 2.5 × 0.2 m. The effective thickness is set as the target deterioration state and periodic observations are made in terms of structural responses under static loading.

#### 4.2.1. System Model: Stochastic Corrosion Model

A stochastic corrosion evolution model introduced by Wang et al. [35] used the system model of the particle filter to represent the underlying corrosion progress. The model was developed based on preconditions including: **a)** corrosion damage is irreversible, **b)** corrosion damage is initially dynamic but eventually will reach a stationary state and **c)** corrosion initiation time and corrosion rate are finite. Considering the stochastic nature of corrosion, the logarithm of corrosion rate at time t, V(t) is described as a Brownian bridge process:

(18a) $$\log\left(V\left(t\right)\right)=\Lambda \left(t\right)+W\left(t\right)-\frac{t-t_{o}}{t_{f}-t_{o}}W\left(t_{f}\right)$$

(18b) $$W\left(t\right)\sim N\left(0,\sigma_{o}^{2}t\right)$$

where W(t) is a generalized Wiener process with parameter $\sigma_{o}$ [36], which describes the uncertainty in the deterioration progress and Λ(t) is the drift term defined as

(19) $$\Lambda \left(t\right)=\ln\left(V_{o}\right)+\left(\alpha -1\right)\log\left(1+\frac{t-t_{o}}{\tau }\right)$$

where $t_{o}$ and $t_{f}$ respectively denote corrosion initiation time and repassivation time (unit: year), $V_{o}$ is the initial corrosion rate (unit: mm/year) and α<1 and τ are positive model constants with no unit. The corrosion depth at time t can be computed by integrating the corrosion rate V(t), that is

(20) $$D\left(t\right)=\int_{0}^{t}V\left(s\right)ds$$

Finally, the effective thickness at each location is evaluated by subtracting the corrosion depth from the initial depth, that is $d\left(t\right)=d_{o}-D\left(t\right)$. In this example, to demonstrate the parameter-updating capability of the proposed PF framework, all parameters including stochastic hyper-parameter $\sigma_{o}$ in Equation (18b), are updated in real-time, as well as the deterioration state, every time structural responses under static loading tests are acquired.

#### 4.2.2. Measurement Model: Inferring Effective Thickness from Measured Structural Responses

**Figure 7:**
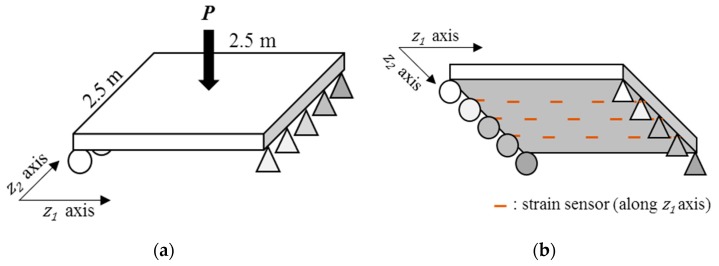
Static loading test setting: (**a**) Loading configurations; (**b**) Sensor configurations (strain).

Suppose strains are measured at the selected locations during periodic static load tests. The configuration of the applied load and sensors are illustrated in Figure 7. For 50 MN of vertical load applied perpendicular to the plate surface, strains are measured from 15 uniformly distributed locations over the surface. The Bayesian inversion model and method proposed in Section 3 is employed as the measurement model of the PF to infer effective thickness from the measured structural responses. 

To obtain the basis function of the spatial pattern of corrosion, the autocovariance function in Equation (17) is used while other types of covariance functions could be introduced without loss of generality. In Equation (17), σ represents the standard deviation of spatial pattern of the effective thickness and is assumed to be 5mm, and $L_{d}=0.9\;m$ is the predefined correlation length.

**Figure 8:**
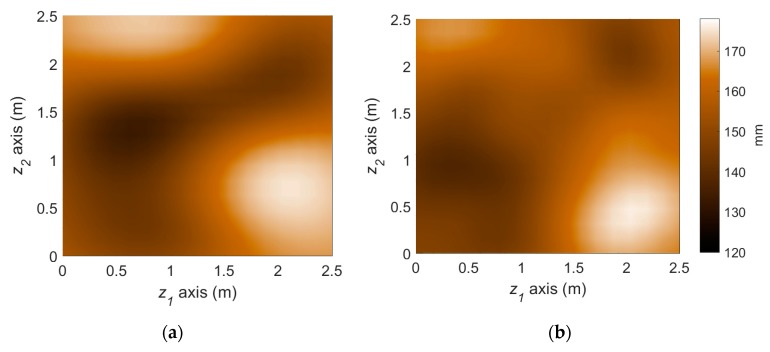
Deterioration state (effective thickness, mm) at year 22 from the reference scenario and Bayesian inversion method: (**a**) Reference deterioration scenario; (**b**) Inferred deterioration pattern.

Figure 8 illustrates the performance of the measurement model, that is Bayesian inversion using KL basis functions. In the finite element simulation performed as the (forward) structural analysis, a total of 289 three-dimensional eight-node solid elements with additional incompatible modes [37] are used to alleviate shear locking issue and reduce the required number of elements. Given the load and sensor configurations provided in Figure 7, the effective thickness of the steel plate is reconstructed with a reasonable level of accuracy by estimating 36 KL-variables, as shown in Figure 8.

#### 4.2.3. Generation of Reference State and Problem Setting

As a reference corrosion scenario, which will be concealed during the numerical verification of the PF applications, a synthetic corrosion depth pattern is generated by a random realization of the growth model in Equations (17) and (18). The reference model parameters are set as $\left\{V_{o},\alpha ,\tau ,\sigma_{o},t_{o}\right\}=\left\{4.8,\;0.15,\;4.0,\;0.2,\;5.0\right\}$ and assumed to be homogeneous along the plate surface. Although the assumed model parameters are homogeneous, the environmental uncertainty induces the spatial variability of corrosion development. While distribution of Equation (18b) is used to generate the environmental uncertainty, spatial correlation in Equation (17) with correlation length of $L_{d}=0.5\;m$ is imposed to emulate the real-world phenomena.

With the reference corrosion states at a certain time instant, the corresponding response strains under the static loading test can be obtained from (forward) structural analysis. The synthetic measurement observation sets are acquired by adding unbiased random noises to the calculated reference strains. Without the loss of generality, it is assumed that repeated measurements are conducted once the sensors are installed, so that the white noises are eliminated and therefore, measurement errors are considered to be trivial (relative error of 0.5%).

#### 4.2.4. Results and Discussions

**Figure 9:**
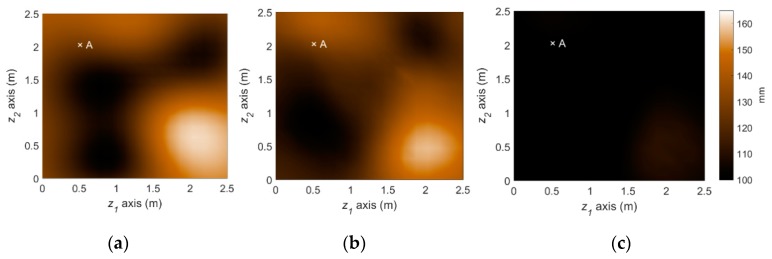
Prediction of deterioration pattern: (**a**) Reference; (**b**) Particle filter with parameter updating (proposed); (**c**) Particle filter without parameter updating.

The reference corrosion pattern at operational year 50 is illustrated in Figure 9a. To simulate the uncertainties in actual conditions of monitoring and prediction, we imposed about 20~50% error for initial knowledge of each parameter. A total of 5 sets of measurements until operational year 22 are assimilated with the stochastic corrosion propagation model using the particle filter framework. The predicted deterioration at year 50 using the proposed method is illustrated in Figure 9b. The spatial pattern of corrosion is captured by the PF-based prediction, allowing us to distinguish highly corroded and less corroded regions. On the other hand, to illuminate the accuracy improvement by the parameter-updating capability of PF, the PF prediction results without parameter updating is also presented in Figure 9c. The use of more inaccurate model parameters leads to significant overestimation in the overall corrosion in this example and gives obscure spatial patterns.

**Figure 10:**
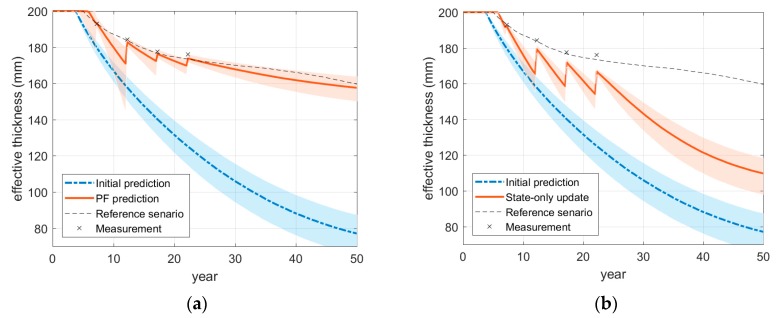
Monitoring and prediction of effective thickness via particle filter: (**a**) Particle filter with parameter updating (proposed); (**b**) Particle filter without parameter updating.

For detailed investigation of monitoring and prediction, Figure 10a shows the time histories of monitoring and prediction of the effective thickness at a point of the plate (point A shown in Figure 9) by the proposed method. The cross-markers in the graphs denote the deterioration states matching the structural response measurements generated from the reference scenario. As more measurements are included, the accuracy of monitoring and prediction of the particle filter gradually improves. When the model parameters are not updated, that is only states are estimated, as shown in Figure 10b, even though the estimation of deterioration is adjusted by measurements, the difference in the propagation trends cannot be reduced effectively compared to the results in Figure 10a.

**Figure 11:**
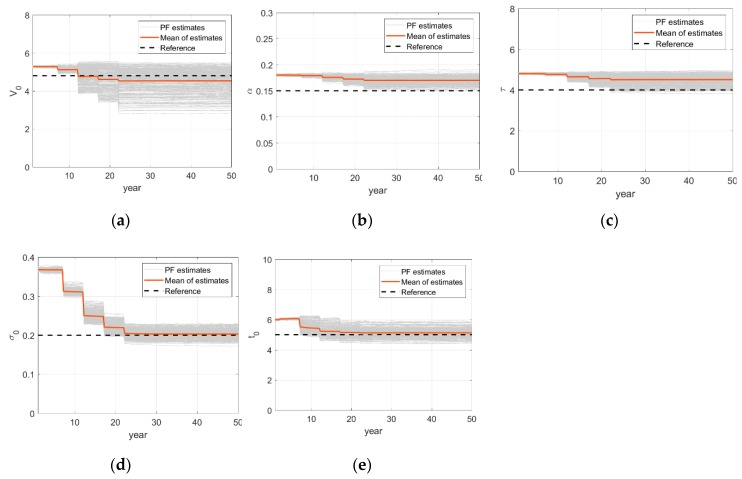
Updating time histories of corrosion model parameters: (**a**) Parameter $V_{o}$; (**b**) Parameter α; (**c**) Parameter τ; (**d**) Parameter $\sigma_{o}$; (**e**) Parameter $t_{o}$.

The PF-based updating histories of deterioration model parameters are shown in Figure 11. The estimations of each parameter at uniformly selected 289 locations and their sample mean are provided along with the reference value. There are some variations for different locations due to environmental uncertainties introduced in the numerical example but the estimates in average seem to approach their reference value. The mean relative errors in estimating hyper parameters, $\left\{V_{o},\alpha ,\tau ,\sigma_{o},t_{o}\right\}$, are respectively {−0.05, 0.13, 0.12, 0.01, 0.02}. One notable discovery is that the stochastic hyper-parameter $\sigma_{o}$, which represents the degree of uncertainty of environmental condition, is also updated accurately by the particle filter.

### 4.3. Prediction of Spatial Corrosion Pattern of Steel Plate with Dynamic Parameters

**Figure 12:**
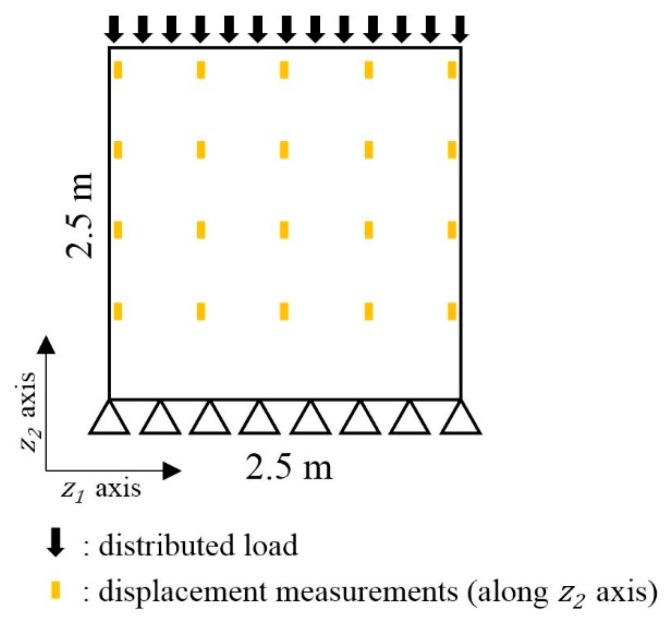
Static loading test setting and displacement sensor locations.

**Figure 13:**
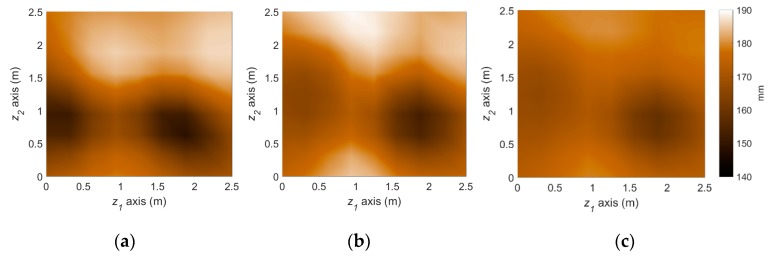
Prediction of deterioration pattern with a dynamic model parameter: (**a**) Reference; (**b**) Particle filter with parameter updating (proposed); (**c**) Particle filter without parameter updating.

**Figure 14:**
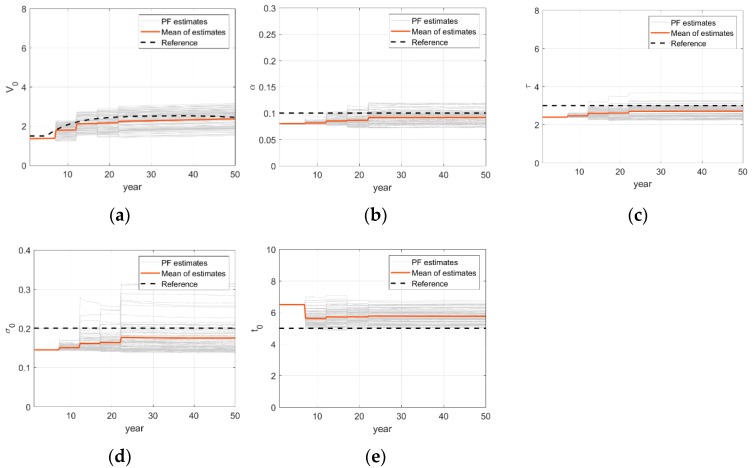
Updating time histories of corrosion model parameters in the presence of a dynamic model parameter: (**a**) Parameter $V_{o}$; (**b**) Parameter α; (**c**) Parameter τ; (**d**) Parameter $\sigma_{o}$; (**e**) Parameter $t_{o}$.

Additional example is considered using the same setting as Section 4.2 but with different type of measurement quantities and load settings. A distributed compression load of 20 MN/m is applied in parallel to the surface and the measurements of *displacement* are considered, as shown in Figure 12. In this example, the reference value of one of the parameters, $V_{o}$, is considered to be dynamic. In detail, the parameter is assumed to be gradually increasing along the operational year with mathematical form of $V_{o}\left(t\right)=V_{o1}+V_{o2}/\left(t-t_{o}\right)\;\mathrm{for}\;t>t_{o}$, where $V_{o1}=1.5$ and $\;V_{o2}=0.2$, while the other model parameters are static: $\;\left\{\alpha ,\tau ,\sigma_{o},t_{o}\right\}=\left\{0.1,\;3,\;0.2,\;5\right\}$. This is an example of situations in which a closed-form mathematical model cannot provide a correct description on corrosion developments. This situation is not unusual considering the complex nature of deteriorations. Figure 13 shows the predicted spatial pattern of deterioration at year 50 and Figure 14 shows the time histories of parameter updating process. It is observed that even if one has not only uncertain parameters but also dynamic parameters, the proposed particle filter can predict spatial pattern of the deterioration with reasonable accuracy. 

## 5. Conclusions

This paper proposes a successive data assimilation method for monitoring and predicting spatiotemporal evolution of structural deterioration. Particle filter approach is used as a primary assimilation algorithm, so that while deterioration transitions are estimated by successive measurements, various uncertain or dynamic parameters involved in the models of deterioration progress are also updated for improved accuracy. Moreover, in order to identify spatially heterogeneous deterioration state based on sparsely distributed measurements of structural responses, a Bayesian inversion method is integrated as a measurement model of the filter. Basis functions of a truncated KL-expansion are adopted to reduce the dimension of the inference domain. From the posterior distribution of a finite number of KL-variables, which are obtained by means of MCMC simulations, the deterioration progress of a continuous structural system is effectively evaluated. By implementing this measurement model into the particle filter approach, a particle filter-based framework predicting spatial pattern of deterioration is constructed.

The proposed method was successfully demonstrated by three numerical examples. The first example aims to monitor and predict corrosion of an embedded reinforcing bar by exploiting explicit non-destructive test measurements regarding the reduced effective cross-sectional area. The result showed that even with homogeneous initial predictions and highly interfered measurements, the heterogeneous aspect of future corrosion can be predicted via the particle filter framework. In the second example, a steel plate element subjected to pitting corrosion is investigated to verify spatiotemporal prognosis performance. At each time point of the periodic inspection under static loading test, the deterioration state is reconstructed via Bayesian inversion method, which works as the measurement model of the particle filter framework. The performance of the proposed method was successfully demonstrated by providing improved monitoring and prediction of spatial distributions of corrosion. The proposed framework can be further applied to a variety of deterioration phenomena including fatigue, given appropriate deterioration progress models. It should be also noted that the proposed approach is expected to be used for the critical local members, rather than the large-scale structures. This is because the global system behavior of proposed mechanical loading-response inspection may not be sensitive to the local deterioration states.

Future research is underway to develop the particle filter for a spatiotemporal reliability analysis. Moreover, this method can be further developed to continuously track the deterioration even under the situation of unexpected or rapid performance degradation, such as natural disasters. The robust performance of the proposed method under stochastic deterioration state can be utilized to build a condition-based maintenance strategy as well.
